# Molecular Dynamics Study of the Swelling of Poly(methyl methacrylate) in Supercritical Carbon Dioxide

**DOI:** 10.3390/ma12203315

**Published:** 2019-10-11

**Authors:** Darya Gurina, Yury Budkov, Mikhail Kiselev

**Affiliations:** 1 G.A. Krestov Institute of Solution Chemistry of the Russian Academy of Sciences, 1 Akademicheskaya St., 153045 Ivanovo, Russia; ybudkov@hse.ru (Y.B.); mgk@isc-ras.ru (M.K.); 2 Tikhonov Moscow Institute of Electronics and Mathematics, National Research University Higher School of Economics, Tallinskayast. 34, 123458 Moscow, Russia

**Keywords:** poly (methyl methacrylate), supercritical carbon dioxide, molecular dynamics, swelling

## Abstract

The swelling of a poly (methyl methacrylate) in supercritical carbon dioxide was studied by means of full atomistic classical molecular dynamics simulation. In order to characterize the polymer swelling, we calculated various properties related to the density, structure, and dynamics of polymer chains as a function of the simulation time, temperature, and pressure. In addition, we compared the properties of the macromolecular chains in supercritical CO_2_ with the properties of the corresponding bulk system at the same temperature and atmospheric pressure. It was shown that diffusion of CO_2_ molecules into the polymer led to a significant increase in the chain mobility and distances between them. Analysis of diffusion coefficients of CO_2_ molecules inside and outside the poly(methyl methacrylate) sample has shown that carbon dioxide actively interacts with the functional groups of poly (methyl methacrylate). Joint analysis of the radial distribution functions obtained from classical molecular dynamics and of the averaging interatomic distances from Car-Parrinello molecular dynamics allows us to make a conclusion about the possibility of formation of weak hydrogen bonds between the carbon dioxide oxygen atom and the hydrogen atoms of the polymer methyl groups.

## 1. Introduction

In recent years, much attention has been paid to the study of polymer-supercritical fluid systems. This is eloquently echoed by a large number of reviews devoted to the use of supercritical carbon dioxide (sc-CO_2_) as a solvent for the physical processing of polymeric materials [1,2], as well as in polymer modification, formation of polymer composites, polymer blending, microcellular foaming, polymerization [3], particle formation [4], and applications of sc-CO_2_ in the fabrication of polymer systems for drug delivery [5]. Being a non-toxic and environmentally friendly solvent, supercritical CO_2_ can replace toxic organic compounds in a number of chemical processes [6,7,8], which is of great interest to the pharmaceutical industry, for example, when creating prolonged dosage forms of drugs by polymer doping [2,9]. The process of polymer impregnation is based on plasticization: when treated in a supercritical solvent, the polymer adsorbs it, which leads to an increase in the mobility of the segments and chains, and an increase in the average distance between the polymer monomers, thereby facilitating the penetration of various additives into the polymer and desorption of undesirable impurities [10,11]. It has been shown previously that CO_2_ is a good plasticizer for amorphous polymers like poly (methyl methacrylate) (PMMA) [12,13]. PMMA/CO_2_ systems were investigated by means of different experimental techniques in a wide range of conditions [14,15,16,17,18,19,20,21,22,23,24]. In particular, sorption and swelling of different polymers including PMMA in the presence of sub- and supercritical carbon dioxide have been measured at pressures up to 30 MPa at 308.2 and 323.2 K in reference [15]. Handa Y.P. and coauthors have studied glass transition in the system PMMA/compressed gas as a function of the gas pressure using high-pressure calorimetric measurements [16]. Sorption of CO_2_ in PMMA at 308−573 K and concurrent dilation of the polymer at 308–358 K over a pressure range up to 5 MPa were studied in reference [18]. Nikitin L.N. and colleges have performed the study of sorption by poly (methyl methacrylate) and poly (butyl methacrylate) in sc-CO_2_ conditions using technique of direct optical observation [19]. In reference [20], a purely gravimetric approach based on the use of a magnetic suspension balance was proposed to simultaneously measure swelling and sorption of supercritical fluids in PMMA in a commercially available setup. Pantoula M. and coauthors used the quartz crystal microbalance and the mass-loss analysis to investigate the sorption of sc-CO_2_ onto PMMA and polystyrene [21], and used the magnetic suspension balance and the optical determination of the volume change to study the swelling process of this polymers [22] under pressures up to 40 MPa and temperatures T = 308–405 K. The authors of reference [25] have experimentally investigated the behavior of PMMA–based systems not only in pure sc-CO_2_, but also in sc-CO_2_ modified by acetone, ethanol, and methylene chloride. They have found that processing PMMA-based polymers with pure sc-CO_2_ leads to polymer swelling, and addition of a liquid cosolvent to CO_2_ enhances polymer dissolution. However, selection of a cosolvent and its concentration is crucial for optimizing solubility. In particular, the polymer solubility in CO_2_ acetone, as a function of cosolvent concentration, reaches a maximum at about 15 wt % of the cosolvent, while for CO_2_-ethanol, the solubility is low and practically not affected by the increase in the cosolvent percentage. In reference [26], the swelling of poly (methyl methacrylate) and poly (butyl methacrylate) bulk samples in supercritical carbon dioxide was studied in situ. The kinetics of swelling, the diffusion coefficients of CO_2_ in the polymers were calculated, the effects of temperature and pressure on the obtained values were analyzed and it was concluded that the degree of PMMA swelling at a fixed exposure temperature (311 K) increased as the pressure grew and showed a nonmonotonic dependence on the temperature.

Along with experimental studies, simulation approaches are useful and powerful tools for investigating systems containing a polymer at the molecular level. Van der Vegt et al. using molecular dynamics (MD) simulations have made calculations of the sorption thermodynamics of CO_2_ in a model glassy polymeric membrane [27] and studied the temperature dependence of carbon dioxide transport in an amorphous polyethylene melt [28]. CO_2_ sorption and swelling in glassy matrices of atactic polystyrene obtained by coarse-graining, equilibration, and reverse-mapping were simulated over the temperatures ranging from 308 to 405 K at pressures of up to 30 MPa [29]. Combining experimental and computational techniques, Zhang et al [30] studied CO_2_-induced plasticization in a polyimide membrane. They discussed the effect of the CO_2_ interactions with the ether groups on the mobility of polyimide chains, calculated the glass transition temperature of the polyimide at different CO_2_ content values in the polymer matrix. In reference [31], the molecular dynamics simulation was used to investigate the adsorption of PMMA and polyvinyl acetate on anα-quartz surface and to understand the interactions between the quartz surface and the polymers. An interesting study of P. Xue et al. [32] focused on the study of the mechanism of a supercritical CO_2_ thickener using MD simulations. The authors examined poly (vinyl acetate-covinyl ether) used as a thickener and showed that adding the polymer reduced the diffusion of supercritical CO_2_ indicating an interaction between the solvent molecules and the polymer functional groups. In particular, it was found that the ester group had better ability to bind a CO_2_ molecule than the ether group.

Although experimental studies of the PMMA swelling process in sc-CO_2_ have already been reported, this phenomenon has not yet been investigated at the microscopic level. To the best of our knowledge, PMMA swelling in sc-CO_2_ has never been studied in detail at the molecular level using MD simulations. It should be noted that the time and length scales of MD simulations are limited to a few nanoseconds and nanometers, and therefore it is not possible to achieve the same system size or observation time as in the experiment. Nevertheless, we can estimate the structural and dynamical changes of the polymer matrix at the molecular level at the very beginning of the swelling process.

We present here a molecular dynamics study of two types of systems: an atactic PMMA in bulk at atmospheric pressure in the temperature interval 273 K–533 K, and PMMA in sc-CO_2_ at temperatures of 333 K and 353 K and pressures 10–25 MPa. We have also calculated and discussed the structural and dynamic characteristics including polymer density, specific volume, coefficient of thermal expansion, glass transition temperature, mean squared displacement, radial distribution functions, gyration radius of the chain, and end-to-end distance.

## 2. Computational Details

**Figure 1:**
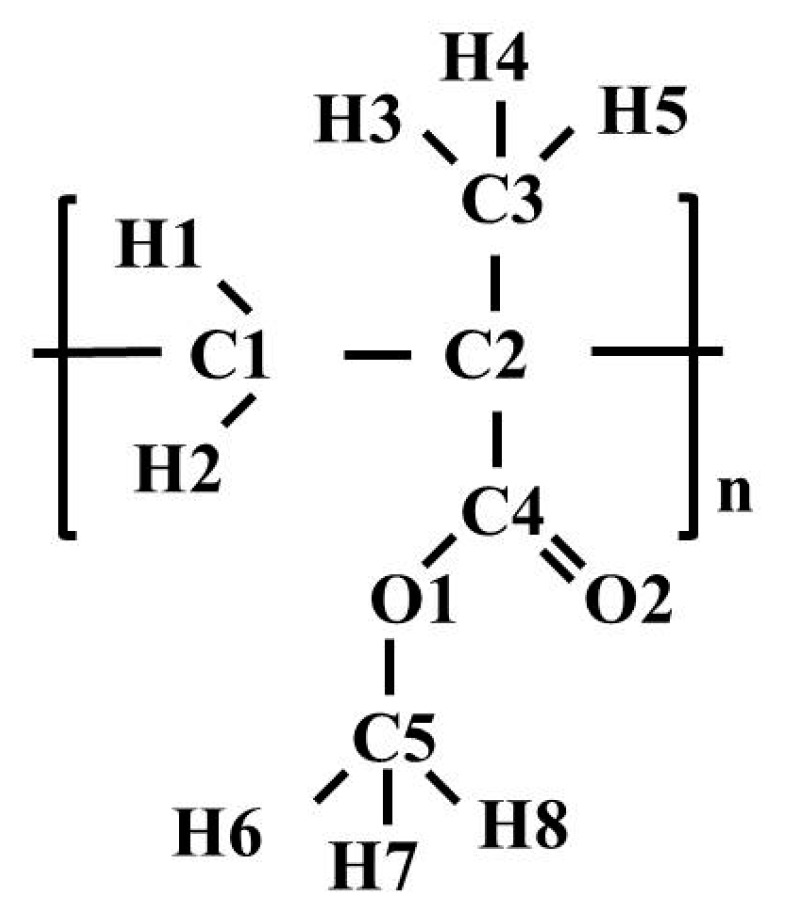
A monomer unit with designations of atoms.

Figure 1 illustrates the monomer unit of PMMA. The polymer chain was built from 100 monomers; then 27 chains were used to construct a three-dimensional structure with an initial density of 1000.0 kg/m^3^ by Materials Studio [33]. As is well known, force field selection is determinant of the accuracy of simulation results of a specific system. In this study, for MD simulations of PMMA in bulk and in sc-CO_2_, the OPLSAA (Optimized Potentials for Liquid Simulations All Atom) force field was used [34,35]. Validation of this force field by comparing the PMMA structure and dynamics with neutron experiments was carried out by C. Chen and co-authors [36]. It was shown that the simulation model provides a fair description of real PMMA samples. The Lennard-Jones (LJ) and partial atomic charge parameters of the OPLSAA force field used for PMMA are given in Table 1. For carbon dioxide, we used the model developed by Z. Zhang and Z. Duan [37]. As for cross site-site interactions, we applied the geometric mean mixing rule for both LJ parameters. The temperature and pressure were controlled by a Nose’-Hoover thermostat [38,39] and a Parrinello-Rahman barostat [40], respectively. The leap-frog integrator was adopted to integrate the equations of motion [41]. The cutoff radius was set of 1.5 nm for all interactions. For the long-range electrostatic interactions, a particle mesh Ewald [42,43] with a grid spacing of 0.25 nm and an interpolation order of four was used. The constraints were implemented using the LINCS algorithm [44]. The time step was 1 fs for all the simulations. In order to gain a homogenous sample of PMMA, the polymer was equilibrated in accordance with the heating and cooling scheme as the one used in a few previous studies [45,46]. All the MD simulations were conducted using GPU-accelerated GROMACS v5.0.7 [47]. Processes of energy minimization, canonical ensemble (NVT, 0.5 ns), and isothermal–isobaric (NPT, 5 ns) ensemble were applied to the PMMA sample at 533 K and 0.1 MPa. After that, the sample was subjected to cooling (for t = 0.5 ns) starting from 533 K and ending at 273 K in ΔT = 10–20 K steps. The equilibrium configurations are performed in NPT ensemble with a pressure of 0.1 MPa and temperature of 273K–533K with 10K–20K steps. At the end of this stage, the densities of the samples and the coefficient of thermal expansion were calculated and compared with the experimental ones [48,49].

In order to investigate the PMMA swelling process, the samples of the polymer equilibrated at 333 K, 353 K, and 0.1 MPa were placed in the center of a cubic cell with periodic boundary conditions and “embedded” by previously equilibrated sc-CO_2_ (85086 molecules) at certain thermodynamic parameters (Table 2). The production run simulations were performed for 10 ns with a time step of 1 fs. The data were collected for analysis every 0.1 ps.

In addition to the classical molecular dynamics, a Car-Parrinello (CPMD) simulation of a small PMMA fragment (consisting of 3 monomer units) in sc-CO_2_has been carried out at T = 333 K and ρ = 725 kg/m^3^ in the CPMD-3.13.2 [51] program package. The total Car-Parrinello dynamical system consists of two adiabatically decoupled subsystems: the cold electronic degrees of freedom and the nuclear degrees of freedom at the relevant physical temperature. The computations have been performed using the gradient-corrected BLYP functional [52,53]. The orbitals of the valence electrons were expanded in a plane wave basis set to a 25 Ry cutoff. The interaction between the core and the valence electrons was described by the ultrasoft pseudopotential in the Vanderbilt form [54]. The Brillouin zone was sampled at the Γ–point only. The fictitious electronic mass and integration step were set up to 600 a.u. and 5 a.u., respectively. The initial configuration consisting of a PMMA fragment surrounded by 58 CO_2_ molecules was simulated by the classical molecular dynamics. The obtained classical trajectory was then equilibrated for 10 ps by means of the CPMD simulation. The production run length was 10 ps; the statistics were gathered every 1.2 fs. The simulation was performed in the NVT ensemble with a Nose–Hoover chain thermostat [38,39].

## 3. Results and Discussion

### 3.1. Bulk PMMA

In order to demonstrate that the force field and simulation parameters were chosen correctly, the polymer physical properties must be realistically represented in the simulation. As it was mentioned in the previous section, the densities of the samples and the coefficient of thermal expansion were calculated and compared with the experimental ones. The results with the theoretical and experimental values, available in the literature [48,49], are reported in Table 3. The densities and coefficients of thermal expansion, obtained for PMMA at different temperatures by means of MD simulations, are in good agreement with the experimental ones.

**Figure 2:**
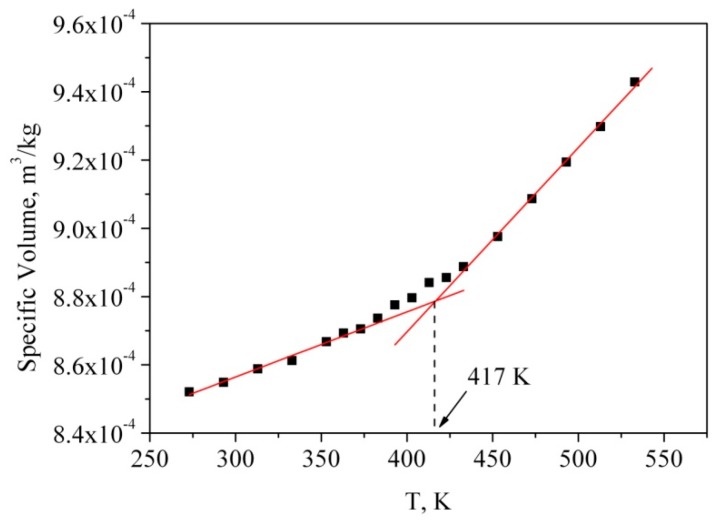
Dependence of specific volume of molecular dynamics (MD) simulation for PMMA.

Glass transition temperature (T_g_) is a unique property of polymers. Below T_g_, polymers behave like glass that is hard and brittle, and above T_g_, polymers act like rubber that is soft and viscous [55,56]. Although polymers possess two completely different states below or above T_g_, glass transition is a second order phase transition, so that many first order properties, for example, volume, change gradually when the temperature increases. Therefore, T_g_ can be derived from fitting the intercept of the two linear trend lines at a low and a high temperature, respectively. There are several ways to estimate T_g_ from MD simulations. In particular, in reference [57] T_g_ was determined through tracing the variations in the macroscopic (thermal conductivity, volume, thermal expansion and Young’s modulus) and microscopic properties (radial distribution functions, mean squared displacement, non-bonded energy) of the polymer during temperature cooling scans. The authors [57] found that the density and volume method was less time consuming in determining the T_g_ than the thermal conductivity and Young’s modulus method. Therefore, we calculated the specific volume, i.e., the inverse density, at each temperature during the cooling process. The temperature dependence of the specific volume of the bulk PMMA is shown in Figure 2.

The temperature gradient of specific volume has a discontinuity at Tg, therefore, the intersection between the lines obtained as interpolation of specific volume values below and above Tg is the estimation of the glass transition temperature. We used this method to obtain the simulated glass transition temperature of the polymer (T_g_ = 417 K). The glass transition temperature value obtained from the simulation was found to be a little higher than the corresponding experimental values (*T_g_ = 378 K [58], T_g_ = 363–387 [59]). These deviations could be caused by the extremely fast cooling rate of the MD simulation (≈10^9^ K/s) relative to the real experiment (≈0.3 K/s) [59]. Moreover, as it was mentioned in reference [57], the T_g_ obtained from MD simulations, is dependent on the polymerization degree, i.e., the higher the polymerization degree is, the higher the glass transition temperature is (T_g_ = 450 K and T_g_ = 381 K for polymerization degree of PMMA 100 and 10, respectively, at a cooling rate 20 K/ns).

Glass transition of polymers is well correlated with mobility of polymer chains, which can be affected by chemical constituents and intermolecular interactions. When a polymer is heated above T_g,_ the intermolecular interactions become weaker and, as a result, the mobility and flexibility of the chains increase. In order to examine the mobility of PMMA during the glass transition process, the mean squared displacement (MSD) was calculated. The MSD curves were calculated by the relation:

(1) $$MSD(t)=\langle {\left|{\vec{r}}_{i}(t)-{\vec{r}}_{i}(t=0)\right|}^{2}\rangle$$

where *r_i_*(*t*) is the position vector of atom *i* at time *t*; the symbol <…> denotes the average for all the atoms as well as for all the time origins.

**Figure 3:**
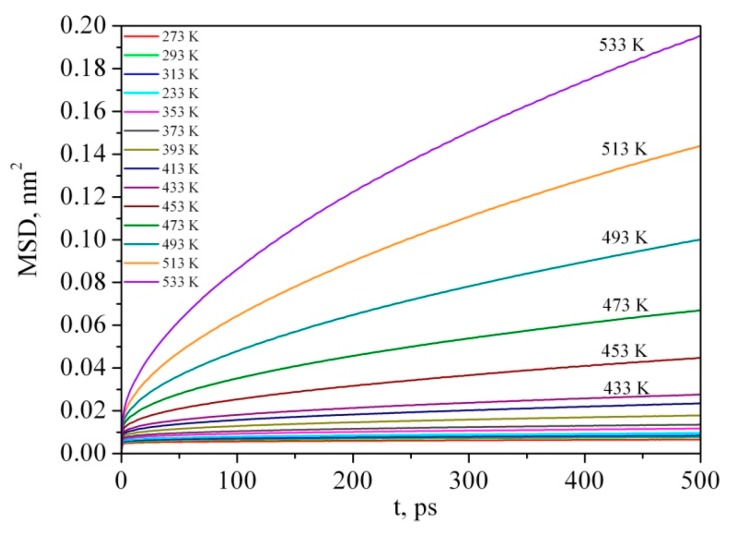
Temperature dependence of mean squared displacement (MSD) curves of PMMA at 0.1 MPa.

The steeper slope of MSD indicates higher mobility of the polymer chain. As one can see in Figure 3, the slopes of MSD above T_g_ are much higher than those below T_g_, showing higher mobility of the polymer chains above the glass transition temperature (namely 417 K).

In reference [57] the same behavior of temperature dependence of MSD curves of PMMA is observed. Namely, the MSD curves remain constant with variation in temperature below 470 K and MSD values notably increased with temperatures raised above 470 K. The difference between our results and data presented in reference [57] is probably due to a small number of PMMA molecules in the MD cell (only 3) which was simulated by M. Mohammadi and coauthors compared with our simulation box containing 27 molecules.

**Figure 4:**
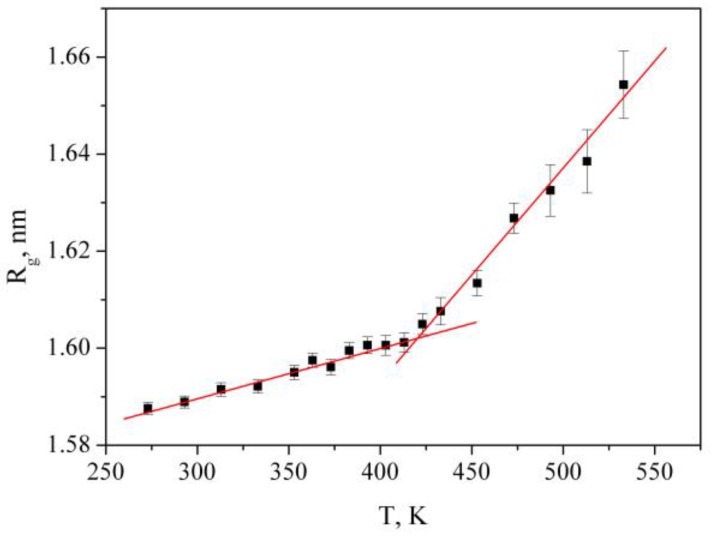
The gyration (*R_g_*) of PMMA in bulk as a function of temperature at 0.1 MPa.

In order to understand how the structural behavior of the polymer samples depends on temperature, we calculated the radius of gyration (*R_g_*), which is one of the most important quantities in conformational statistics of polymer chains [60,61,62]. The radius of gyration was calculated by the following Equation:

(2) $$R_{g}={\left(\frac{{\sum_{i}\left\|r_{i}\right\|}^{2}m_{i}}{\sum_{i}m_{i}}\right)}^{1/2}$$

where *m_i_* is the mass of site *i* and *r_i_* is the position of site *i* relative to the center of mass of the molecule. As can be seen from Figure 4, there is an abrupt change in the slope of *R_g_* as a function of temperature around 417 K. Thus, at temperatures above 417 K, significant changes in the structure of the polymer are observed, i.e., the polymer transitions from a glassy state to a highly elastic state. It should be noted that the Tg value obtained from the temperature dependence of the specific volume of the bulk PMMA coincides with the value obtained from *R_g_*(T).

### 3.2. PMMA in sc-CO_2_

**Figure 5:**
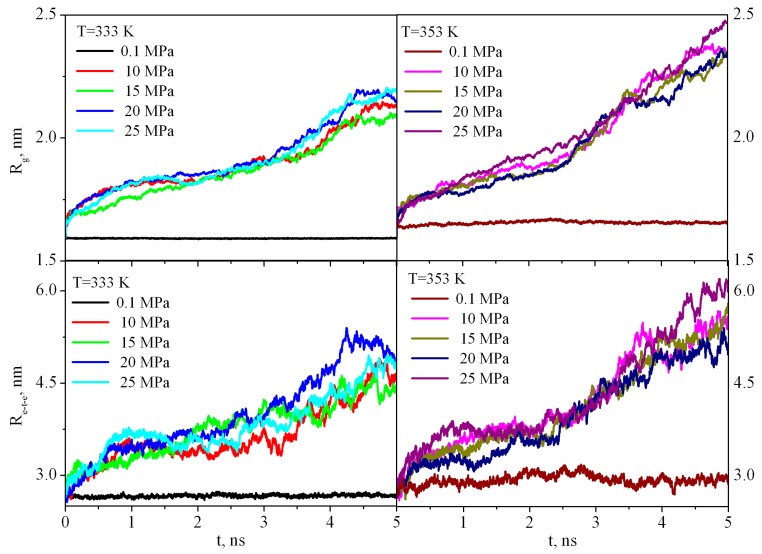
Dependence of the radius of gyration (*R_g_*) and end-to-end distance (*Re-t-e*) of PMMA in sc-CO_2_ at pressures of 10-25 MPa and in the bulk at 0.1 MPa during the first 5 ns of the simulation.

The first stage of dissolution of any polymer is its swelling. Swelling is the process of absorption of a low molecular weight solvent by a polymer, accompanied by an increase in the mass and volume of the polymer and a change in the conformation of its macromolecules. Because the PMMA solubility is negligible in sc-CO_2_ (it is less than 0.001 percentile weight of the extracted amount per unit mass of CO_2_ at 333 K and 20 MPa) [25], the dissolution process stops at the stage of swelling, and, thus, one can discuss limited swelling of PMMA in supercritical carbon dioxide. PMMA swelling in sc-CO_2_leads to an increase in the end-to-end distance R_ete_ and radius of gyration *R_g_* (Figure 5) in comparison with the bulk PMMA where R_ete_ and *R_g_* fluctuate around a constant value during the entire simulation time.

In order to investigate the mobility of PMMA segments, we calculated the self-diffusion coefficients of the PMMA from the slope of the MSD according to the Einstein’s relation [63] as follows:

(3) $$D=\frac{1}{6}\underset{t\to \infty }{\lim}\frac{dMSD}{dt}$$

As Table 4 shows, the PMMA swelling in CO_2_ changes the mobility of the chains. The self-diffusion coefficient of the polymer in the supercritical solvent is three orders of magnitude higher than in the bulk.

**Figure 6:**
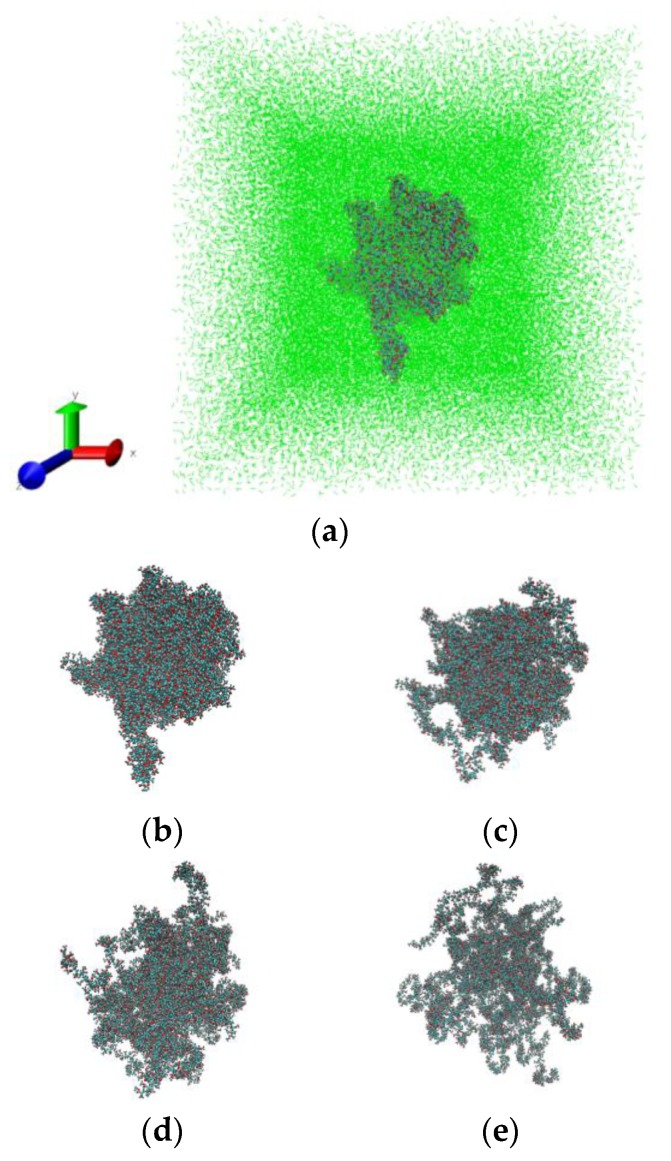
Snapshots of PMMA in sc-CO_2_ (green) at 333 K and 25 MPa: 0 ns (**a**,**b**), 2 ns (**c**), 5 ns (**d**) and 10 ns (**e**). The solvent molecules have been deleted from figures (**b**–**e**) for clarity to highlight polymer swelling.

PMMA in sc-CO_2_ has comparatively higher mobility, and thus requires a lower temperature compared with the bulk PMMA to obtain the same segment mobility. The increase in mobility of the polymer molecules after adding CO_2_ can be understood as a plasticization effect due to the increased space between the chain segments. Figure 6 illustrates the swelling process of PMMA in sc-CO_2_ at 333 K and 25 MPa. The snapshots have been made by using the visualization program VMD [64].

**Figure 7:**
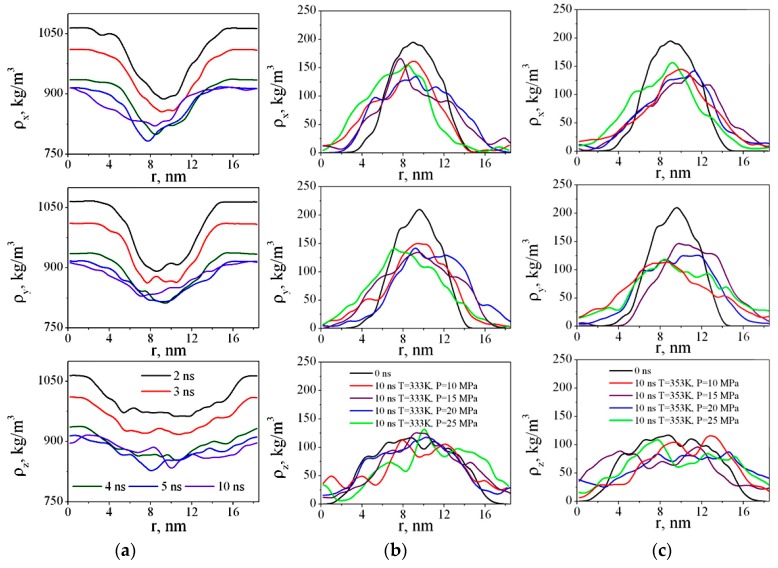
Time dependences of CO_2_ density in simulation boxes in x, y, and z directions at 333 K and 25 MPa (**a**), PMMA densities in x, y, and z directions at the beginning and at the end of the simulation at P = 10–25 MPa and 333 K (**b**), 353 K (**c**).

We have calculated the density of CO_2_ and PMMA in x, y, and z directions for each nanosecond (Figure 7).

Figure 7a shows dependences ρ_x_(r), ρ_y_(r), and ρ_z_(r) after 2, 3, 4, 5, and 10 ns. Figure 7b,c show the density profiles of PMMA versus time during polymer swelling in sc-CO_2_. As we can see, in the first four nanoseconds after the beginning of the simulation, more dramatic changes in the PMMA structure are observed. The CO_2_ molecules gradually diffuse into the polymer and the difference in the solvent density at the edges of the box (i.e., outside the polymer) and closer to the center of the box (i.e., in the area where the polymer is located) decreases. The density profiles of PMMA become wider and lower (Figure 7b,c), meaning the polymer adsorbs the solvent molecules and swells.

The ability of polymers to swell is characterized by the degree of swelling (α), which is defined as the amount of solvent absorbed by the polymer, per unit mass or volume of the polymer:

(4) $$\alpha =\frac{m-m_{0}}{m_{0}}=\frac{(m_{0}+m_{ads})-m_{0}}{m_{0}}=\frac{m_{ads}}{m_{0}}$$

(5) $$\alpha =\frac{V-V_{0}}{V_{0}}$$

where *m*_0_ and *V*_0_ are the mass and volume of the original polymer, respectively; *m* and *V* are, respectively, the mass and volume of the swollen polymer, and *m_ads_* is the mass of the solvent adsorbed by the polymer. We try to estimate the degree of PMMA swelling by using the first method, namely, focusing on the mass. The calculation was carried out as follows: the mass of carbon dioxide molecules, located at a distance of 0.5 nm from any PMMA atom was calculated at the initial time (*m*_0_(CO_2_)) and every ns (*m_t_*(CO_2_)). Then we found the change in the mass of carbon dioxide near PMMA, compared to the initial value: *m_ads_*(CO_2_) = *m_t_*(CO_2_)–*m*_0_(CO_2_), i.e., the mass of the solvent adsorbed by the polymer, and then used Equation (4).

Limited swelling occurs over a long time and is determined by the rate of diffusion of the solvent molecules into the polymer. In the simplest case, the swelling process proceeds as a first-order reaction, therefore the swelling rate is equal to:

(6) $$\frac{d\alpha }{dt}=k(\alpha_{\max}-\alpha )$$

where *k* is the swelling rate constant, *α*_max_ is the maximum degree of swelling.

Thus, the swelling kinetics Equation has the following form:

(7) $$\alpha_{t}=\alpha_{\max}(1-e^{-kt})$$

**Figure 8:**
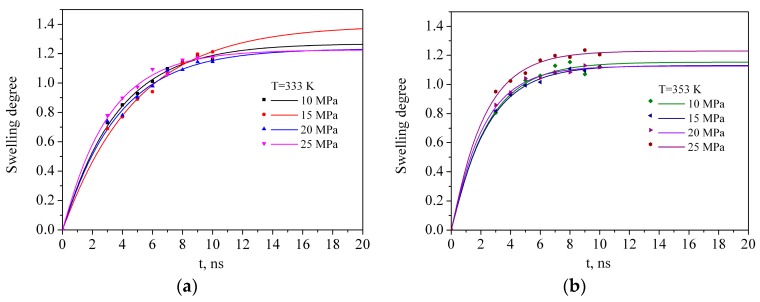
Dependence of polymer swelling degree (symbols) and theoretical approximations of the MD data according to Equations (7) (lines) at 333 K (**a**) and at 353 K (**b**).

Using Equation (7), the degree of PMMA swelling obtained from MD by means of Equation (4) was described as a function of time (Figure 8). The standard deviation was about 0.99. Table 5 shows the coefficients of the swelling equation for all of the systems.

Certainly, the maximum degree of PMMA swelling in sc-CO_2_obtained from MD simulations is significantly different from the experimental results. For example, in reference [64,65,66] it has been shown that the equilibrium degree of swelling of PMMA in sc-CO_2_ can be as high as 20 wt %, and the authors of reference [26] obtained a volume degree of PMMA swelling of 32 ± 6% at 323K and 12.5 MPa and 37 ± 5% at 311 K and 12.5 MPa. In the recent work of R. Li et al. [24] the swelling ratio, which is defined as the ratio of the volume change under isobaric conditions during the swelling with the initial volume of the polymer sample at ambient temperatures, was found to be 0.8654 at 353 K and 10 MPa and the highest value of the swelling ratio 1.1910 was achieved at 363 K and 12 MPa. Such discrepancies between the MD and the experiment are the result of differences in the sample molecular weight and size: in reference [26] the molecular weight of PMMA was M_W_ = 4098 kDa, in reference [24] the average molecular weight was 500 kDa, while in the MD simulations it was a few orders of magnitude lower than in the experiments, M_W_ = 10 kDa. Based on the analysis of the data presented in Table 5, it can be concluded that temperature has a positive effect on the PMMA swelling rate, but has a negative effect on the maximum swelling degree of the polymer, which is probably more highly influenced by the density of the solvent rather than by the temperature or pressure. Based on experimental study of swelling and impregnation process of PMMA in supercritical CO_2_, the authors of reference [67] have concluded that volume expansion of PMMA increases with increase in pressure and decreases with increases in temperature. Moreover, the effects of pressure and temperature on the extent of volume increase were directly related to the increase in solvent density.

The addition of a PMMA sample to sc-CO_2_ decreases the diffusion of the CO_2_ molecules compared to the pure fluid, which indicates that carbon dioxide actively interacts with the PMMA functional groups (Table 6). These data clearly show that the diffusion coefficients of the CO_2_ molecules which are located inside the PMMA sample near the ester groups are 1.5–2 times lower than the system averaged diffusion coefficients of the CO_2_ molecules, and even up to 8 times lower than in bulk supercritical carbon dioxide. Among other things, the latter fact demonstrates the polymer thickening ability in relation to the solvent. One can see that while in the pure supercritical carbon dioxide, the CO_2_ diffusion coefficients are strongly dependent on the pressure (or, in other words, on the solvent density) and vary from ≈23 × 10^−5^ cm^2^/s to ≈111 × 10^−5^ cm^2^/s, in the mixture with the polymer, the diffusion coefficients values of the solvent molecules vary around one order (10–14) × 10^−5^ cm^2^/s. CO_2_ diffusion inside PMMA does not almost depend on temperature and pressure and D_CO2_ values vary from ≈6 × 10^−5^ cm^2^/s to ≈7 × 10^−5^ cm^2^/s. The latter may indicate that internal structure of PMMA samples (for example, size of cavities where CO_2_ molecules are located) is similar. Through analysis of the movement of optical boundaries and the kinetics of swelling, the diffusion coefficients of CO_2_ in the PMMA were calculated by Gallyamov M. O. and colleagues [26]. For instance, they found that at 311 K and 15 MPa, the diffusion coefficients of CO_2_ obtained with using optical technique and from volume swelling kinetics are equal at (0.20 ± 0.03) × 10^−5^ cm^2^/s and (0.07 ± 0.03) × 10^−5^ cm^2^/s, respectively. Taking into account the differences in the temperature and the sample size in the experiment and our simulations, we can conclude that the diffusion coefficients from MD simulations are consistent with the experimental data. Gallyamov M. O. and colleagues [26] also noted that as the temperature increased from 311 K to 338 K, the diffusion coefficient in PMMA increased by 20%–40%. In our case when the temperature increased from 333 K to 353 K, the diffusion coefficient in PMMA-CO_2_ system also increased by 20%–27% depending on the pressure.

**Figure 9:**
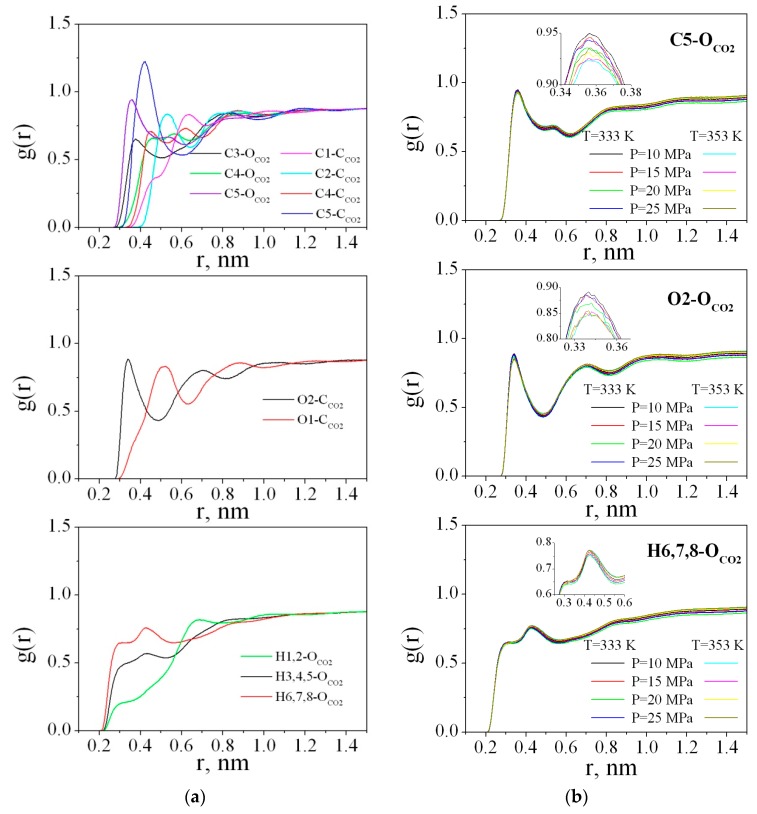
Radial distribution functions of PMMA and CO_2_ atoms: (**a**) at 333 K and 25 MPa; (**b**) C5-O_CO2_, O2-C_CO2_, and H6,7,8-O_CO2_ for all thermodynamic states.

The radial distribution functions (RDFs) between the PMMA and CO_2_ atoms were obtained by averaging over the last 2 ns of the trajectory. Figure 9a illustrates the RDFs g (r) (C1-C_CO2_, C2-C_CO2_, C3-O_CO2_, C4-C_CO2_, C4-O_CO2_, C5-C_CO2_, C5-O_CO2_, O1-C_CO2_, O2-C_CO2_, H1,2- O_CO2_, H3,4,5- O_CO2_, and H6,7,8- O_CO2_) for the polymer in sc-CO_2_ at 333 K and 25 MPa. The RDFs for the other states behave in the same way and Figure 9b presents only the most pronounced RDFs, namely, C5-O_CO2_, O2-C_CO2_, and H6,7,8- O_CO2_ for all the thermodynamic points.

As clearly shown at Figure 9a, in the range of r < 0.5 nm, peaks on the RDFs carbon(PMMA)-oxygen(CO_2_)/carbon(CO_2_) are well distinguished. These peaks determine the arrangement of solvent molecules around the polymer. The most pronounced and highest peaks on the RDFs C5-C_CO2_, C5-O_CO2_, O2-C_CO2_, and H6,7,8-O_CO2_ compared to each other allow us to conclude that the ester groups of the polymer are more solvated by carbon dioxide than the methyl groups (including C3, H3, H4, H5 atoms), as well as the carbon and hydrogen atoms of the chain (C1, C2, H1, H2). In the literature [68], it has been found that there are specific interactions between CO_2_ and PMMA which are most probably of a Lewis acid-base nature. However, the strength of such a specific interaction is very weak. Actually the first peak on the O2-C_CO2_ RDF from 0.3 to 0.5 nm could be attributed to electron donor-acceptor (EDA) interactions because the maximum is located around 0.35 nm and is within the geometric criterion (0.26 ≤ R_C--O_ ≤ 0.43 nm) for EDA interactions used by Xu W. et al. and Saharay M. et al. [69,70]. For the O1-C_CO2_ RDF, the first peak is wider and shifted to 0.5 nm and the small shoulder located in the range 0.3–0.43 nm indicates low probability of O1 atom participation in the EDA interactions with the CO_2_ molecules.

**Figure 10:**
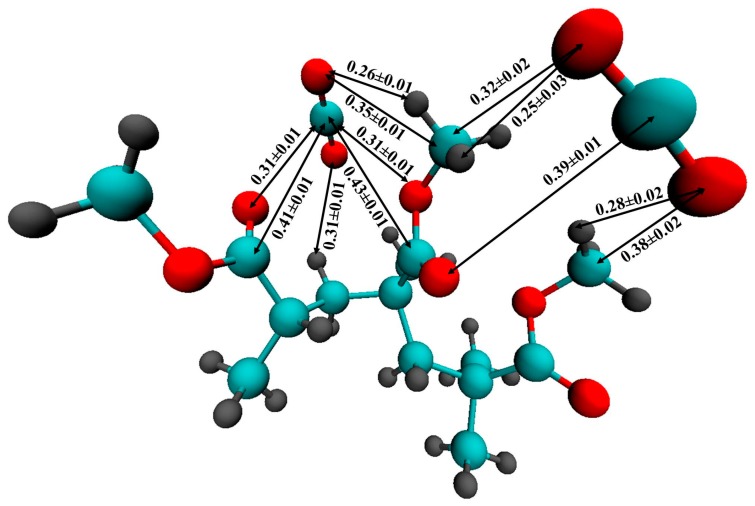
The complex of a PMMA fragment with two CO_2_ molecules according to the CPMD. All the distances are given in nm. The averaging of the distances was made over the last 120 fs. (The red spheres denote the oxygen atoms, the gray ones represent the hydrogen atoms, the cyan ones represent the carbon atoms).

For a more accurate description of the intermolecular interaction between CO_2_ and PMMA, we carried out an ab initio Car-Parrinello molecular dynamics simulation of a PMMA fragment consisting of 3 monomer units surrounded by 58 CO_2_ molecules. Figure 10 illustrates the mutual arrangement of two CO_2_ molecules interacting with the polymer fragment at the end of the simulation. Visual inspection of the CPMD trajectory shows that such a triple complex existed for ≈120 fs. It is interesting to note that the main distances between the atoms of PMMA and CO_2_ are in agreement with the positions of the peaks on the respective RDFs obtained by classical MD. In Figure 10, one can see that the carbon dioxide molecules are oriented in such a way that they can interact with the hydrogen atoms of the PMMA methyl groups. Previously, P. Raveendran and S. L. Wallen [71] investigated the role of cooperative C-H⋯O hydrogen bonds (HBs) as a stabilization factor in addition to the EDA interactions between CO_2_ and carbonyl group by using ab initio calculations at the second-order Møller-Plesset (MP2) [72,73] level. Despite the fact that the C-H⋯O hydrogen bonds (HBs) are very weak, they may have an important stabilizing effect. Assuming that the interaction between the PMMA O atoms and the CO_2_C atoms is the only EDA type, it can be expected that two C=O bond lengths of CO_2_ should be identical. However, the asymmetry of the C = O bonds in the CO_2_ molecules (1.15 Å and 1.19 Å, 1.17 Å and 1.20 Å for two CO_2_ molecules, respectively) and, in addition, the shortening of the C-H bonds participating in the HBs (1.09 Å) compared with “free” C-H (1.14-1.16Å) allow us to suppose that there is interaction like an HB. Moreover, the distance between C of the methyl groups and O of CO_2_ is less than the upper limit (0.4 nm) for C-H⋯O hydrogen bonding [74]. On the classical RDFs C5-O_CO2_, and H6,7,8-O_CO2_, the peaks are located at 0.35 and 0.28 nm, respectively, which is also within the geometric criterion of weak C-H⋯O HB.

For PMMA, the research of J. R. Fried and W. Li [75] indicated that there are weak dipole-dipole interactions between the ester moieties in PMMA. Also, they demonstrated that carbon dioxide is capable of overcoming the internal interactions of the carbonyl groups. Our findings concerning specific interactions between CO_2_ and PMMA confirm that they are responsible for the observed plasticization of the polymer.

## 4. Conclusions

The simulations performed on bulk PMMA at atmospheric pressure have been used to validate the model and the force field employed in the classical molecular dynamics simulations. The simulations were able to satisfactorily reproduce PMMA’s experimental density, glass transition temperature and coefficient of thermal expansion. An equilibrated PMMA sample was then placed in a sc-CO_2_ medium in different thermodynamic states. In comparison with bulk PMMA, we observed an increase in the end-to-end distance and radius of gyration, following PMMA swelling in sc-CO_2_. Moreover, in the sc-CO_2_ medium, the chains mobility and the distance between them were significantly increased. The kinetics of polymer swelling has also been studied. The results have shown that the PMMA swelling degree is overestimated compared to the experimental values due to the limitations inherent in MD simulations, which are related to the size of the polymer sample. Nevertheless, the present calculations demonstrate that the combined use of the classical and ab initio MD provides a good way to better understand the intermolecular interactions in the polymer/fluid system. In particular, the PMMA intermolecular interactions with the solvent molecules are not only of the electron donor-acceptor type but also weak C-H⋯O hydrogen bonds. The results of MD indicate that sc-CO_2_ could be a desirable swelling agent in the impregnation of PMMA with additives and, therefore, we will make the molecular mechanism of the diffusion of small organic molecules into the polymer matrix in supercritical media the subject of further publications.

## Figures and Tables

**Table 1:** Energy parameters and partial atomic charges for used potential model of poly (methyl methacrylate) (PMMA).

Atom	σ, nm	ε, kJ mol^−1^	q, e.c.
C1	0.350	0.28	−0.09
C2	0.350	0.28	0.00
C3	0.350	0.28	−0.135
C4	0.375	0.44	0.51
C5	0.350	0.28	0.16
O1	0.300	0.71	−0.33
O2	0.296	0.88	−0.43
H1, H2	0.250	0.13	0.045
H3, H4, H5	0.250	0.13	0.045
H6, H7, H8	0.242	0.06	0.03

**Table 2:** Stated parameters of the simulated systems: Temperature T, Pressure P, experimental Density of CO_2_ ρ(CO_2_)_EXP_ [50], total Density of the systems at the end of simulation ρ_MD_, and size of cubic cell L.

System	T, K	P, MPa	ρ(CO_2_) _EXP_., kg/m^3^	ρ_MD_, kg/m^3^	L, nm
1	333	10	290.81	913.99	19.4
2		15	605.60	924.57	19.3
3		20	724.63	933.42	19.3
4		25	787.28	942.28	19.2
5	353	10	221.93	856.48	19.8
6		15	428.15	870.73	19.7
7		20	594.85	888.32	19.6
8		25	686.98	904.38	19.4

**Table 3:** Comparison of properties (density ρ and coefficient of thermal expansion β) at atmospheric pressure in the temperature range 273 – 533 K obtained by using MD and experimentally.

T, K	ρ, kg/m^3^	ρ_exp_, kg/m^3^	β, 10^−4^ 1/K	β_exp_, 10^−4^ 1/K
273	1174 ± 2	1175 [48]	1.62	
293	1170 ± 2	1170 [48]	2.13	
303	1167 ± 2		2.29	
313	1164 ± 2		1.67	1.80 [49]
333	1161 ± 2	1160 [48]	2.28	2.10 [49]
353	1154 ± 2	1155 [48]	3.01	2.40 [49]
363	1150 ± 3		2.12	
373	1149 ± 3	1150 [48]	2.43	2.70 [49]
383	1145 ± 3		3.92	
393	1140 ± 3	1140 [48]	3.34	5.50 [49]
403	1137 ± 3		3.58	
413	1131 ± 3	1128 [48]	3.24	5.80 [49]
423	1129 ± 5		2.53	
433	1125 ± 3	1126 [49]	4.07	6.10 [49]
453	1114 ± 4	1112 [49]	5.26	6.40 [49]
473	1101 ± 4	1097 [49]	5.63	6.70 [49]
493	1088 ± 5	1082 [49]	5.33	7.00 [49]
513	1076 ± 5	1067 [49]	5.77	7.20 [49]
533	1061 ± 5	1052 [49]	6.36	7.50 [49]

**Table 4:** Self-diffusion coefficients of PMMA in bulk and in sc-CO_2_.

System	T, K	P, MPa	D, 10^−5^ cm^2^/s
Bulk PMMA	333	0.1	0.0004 ± 0.0001
PMMA in sc-CO_2_		10	0.89 ± 0.09
		15	0.75 ± 0.04
		20	0.75 ± 0.04
		25	0.68 ± 0.10
Bulk PMMA	353	0.1	0.0005 ± 0.0002
PMMA in sc-CO_2_		10	1.07 ± 0.11
		15	0.91 ± 0.12
		20	0.85 ± 0.12
		25	0.78 ± 0.15

**Table 5:** The rate (*α*_max_ - the maximum degree of swelling, *k* - the swelling rate constant) of swelling from Equation (7) for all thermodynamic points. The numbers after ± denote the standard deviation.

T, K	P, MPa	α_max_	k
333	10	1.27 ± 0.02	0.28 ± 0.01
	15	1.39 ± 0.05	0.21 ± 0.02
	20	1.23 ± 0.04	0.27 ± 0.02
	25	1.23 ± 0.02	0.33 ± 0.02
353	10	1.15 ± 0.02	0.42 ± 0.03
	15	1.13 ± 0.01	0.42 ± 0.01
	20	1.13 ± 0.01	0.47 ± 0.02
	25	1.23 ± 0.01	0.47 ± 0.02

**Table 6:** Diffusion coefficient of CO_2_ inside PMMA, in the PMMA-CO_2_ system, and in pure sc-CO_2_.

T, K	P, MPa	D_CO2_ (inside PMMA), 10^−5^ cm^2^/s	D_CO2_ (in PMMA-CO_2_ system), 10^−5^ cm^2^/s	D_CO2_ (in pure CO_2_), 10^−5^ cm^2^/s
333	10	7.0 ± 2.1	11.5 ± 0.1	76 ± 3
333	15	6.4 ± 0.6	11.1 ± 0.3	31 ± 2
333	20	6.2 ± 0.2	10.81 ± 0.04	23.5 ± 0.8
333	25	6.4 ± 0.2	10.3 ± 0.1	23 ± 1
353	10	7.2 ± 0.9	14.66 ± 0.01	111 ± 9
353	15	6.8 ± 0.5	13.93 ± 0.07	52 ± 2
353	20	7.2 ± 1.0	13.3 ± 0.2	32.3 ± 0.4
353	25	6.2 ± 0.3	12.4 ± 0.1	26.2 ± 0.7
